# Analgesic efficacy and safety of morphine in the Procedural Pain in Premature Infants (Poppi) study: randomised placebo-controlled trial

**DOI:** 10.1016/S0140-6736(18)31813-0

**Published:** 2018-12-15

**Authors:** Caroline Hartley, Fiona Moultrie, Amy Hoskin, Gabrielle Green, Vaneesha Monk, Jennifer L Bell, Andrew R King, Miranda Buckle, Marianne van der Vaart, Deniz Gursul, Sezgi Goksan, Edmund Juszczak, Jane E Norman, Richard Rogers, Chetan Patel, Eleri Adams, Rebeccah Slater

**Affiliations:** a Department of Paediatrics, University of Oxford, Oxford, UK; b National Perinatal Epidemiology Unit, Nuffield Department of Population Health, University of Oxford, Oxford, UK; c Tommy's Centre for Maternal and Fetal Health, MRC Centre for Reproductive Health, Queen's Medical Research Institute, University of Edinburgh, Edinburgh, UK; d Department of Anaesthetics, John Radcliffe Hospital, Oxford University Hospitals NHS Foundation Trust, Oxford, UK; e Department of Ophthalmology, John Radcliffe Hospital, Oxford University Hospitals NHS Foundation Trust, Oxford, UK; f Newborn Care Unit, John Radcliffe Hospital, Oxford University Hospitals NHS Foundation Trust, Oxford, UK

## Abstract

**Background:**

Infant pain has immediate and long-term effects but is undertreated because of a paucity of evidence-based analgesics. Although morphine is often used to sedate ventilated infants, its analgesic efficacy is unclear. We aimed to establish whether oral morphine could provide effective and safe analgesia in non-ventilated premature infants for acute procedural pain.

**Methods:**

In this single-centre masked trial, 31 infants at the John Radcliffe Hospital, Oxford, UK, were randomly allocated using a web-based facility with a minimisation algorithm to either 100 μg/kg oral morphine sulphate or placebo 1 h before a clinically required heel lance and retinopathy of prematurity screening examination, on the same occasion. Eligible infants were born prematurely at less than 32 weeks' gestation or with a birthweight lower than 1501 g and had a gestational age of 34–42 weeks at the time of the study. The co-primary outcome measures were the Premature Infant Pain Profile–Revised (PIPP-R) score after retinopathy of prematurity screening and the magnitude of noxious-evoked brain activity after heel lancing. Secondary outcome measures assessed physiological stability and safety. This trial is registered with the European Clinical Trials Database (number 2014-003237-25).

**Findings:**

Between Oct 30, 2016, and Nov 17, 2017, 15 infants were randomly allocated to morphine and 16 to placebo; one infant assigned placebo was withdrawn from the study before monitoring began. The predefined stopping boundary was crossed, and trial recruitment stopped because of profound respiratory adverse effects of morphine without suggestion of analgesic efficacy. None of the co-primary outcome measures differed significantly between groups. PIPP-R score after retinopathy of prematurity screening was mean 11·1 (SD 3·2) with morphine and 10·5 (3·4) with placebo (mean difference 0·5, 95% CI −2·0 to 3·0; p=0·66). Noxious-evoked brain activity after heel lancing was median 0·99 (IQR 0·40–1·56) with morphine and 0·75 (0·33–1·22) with placebo (median difference 0·25, 95% CI −0·16 to 0·80; p=0·25).

**Interpretation:**

Administration of oral morphine (100 μg/kg) to non-ventilated premature infants has the potential for harm without analgesic efficacy. We do not recommend oral morphine for retinopathy of prematurity screening and strongly advise caution if considering its use for other acute painful procedures in non-ventilated premature infants.

**Funding:**

Wellcome Trust and National Institute for Health Research.

## Introduction

Premature infants (born before 37 weeks' gestation) in neonatal intensive care undergo frequent painful procedures without adequate pain relief.^1^ Analgesic options are limited because few clinical trials have been done in infants^2^ and efficacy and safety data are frequently inconclusive.^3^ Eye examinations to detect retinopathy of prematurity—a retinal vascular disorder that can cause permanent blindness if untreated^4^—have reduced significantly the risk of visual impairment in premature infants,^5^ but this examination is painful, stressful, and causes substantial physiological instability for 48 h post procedure.^6^, ^7^, ^8^ Current pain management options—including sucrose, local anaesthetic eye drops, breast milk, and comfort measures (eg, containment holding)—are effective for minor procedures but are not thought to provide adequate pain relief for retinopathy of prematurity screening^9^ and do not prevent the physiological instability caused by the procedure.^8^ An analgesic is needed that can be administered safely to provide effective procedural pain relief to premature infants, regardless of intravenous access, care setting, or ventilatory status.

Intravenous morphine is frequently used for sedation during invasive ventilation in neonates, but evidence for its analgesic efficacy remains contradictory and inconclusive.^3^ Pain assessment is challenging in the absence of verbal report, and composite behavioural and physiological pain scores have formed the cornerstone of infant pain assessment.^10^ Recently, neurophysiological measures have been developed to objectively quantify pain-related brain activity and reflex activity; these measures have been well characterised after heel lancing.^11^, ^12^

**Research in context**

**Evidence before this study**

Morphine is one of the most frequently prescribed analgesics in neonatal practice. Although evidence suggests that intravenous morphine provides sedation in ventilated infants, and some research suggests it might provide effective analgesia for acute painful procedures (eg, chest drain insertion and central line placement), differences in study designs, dosing, heterogeneity of outcome measures, and administration of rescue boluses have made interpretation of the evidence challenging. A Cochrane review reported that evidence is insufficient to recommend routine clinical use of morphine for procedural pain relief in ventilated infants. Many neonatal formularies include oral morphine as a treatment for pain in neonates, and suggested doses are 50–200 μg/kg. A previous pilot study investigated the analgesic efficacy of oral morphine before retinopathy of prematurity screening, but findings were inconclusive because the study was stopped early owing to changes in Medicines and Healthcare products Regulatory Agency regulations.

**Added value of this study**

In this study, 100 μg/kg oral morphine was administered to non-ventilated infants before retinopathy of prematurity screening and clinical heel lancing. Multiple modalities were used to quantify analgesic efficacy, which included changes in pain scores, noxious-evoked brain activity, and reflex withdrawal activity. A comprehensive approach was used to assess changes in oxygen saturation, respiratory rate, heart rate, and ventilation requirement in the 24 h period before and after the clinical procedures. In our study, administration of 100 μg/kg oral morphine in non-ventilated premature infants had profound respiratory adverse effects without suggestion of analgesic efficacy.

**Implications of all the available evidence**

We do not recommend the use of oral morphine at a dose of 100 μg/kg in non-ventilated premature infants for retinopathy of prematurity screening. Morphine produces cardiorespiratory effects that last for an average of 6–8 h. If this dose or a greater dose of morphine were to be administered to infants for other clinical indications or in future clinical trials, the infant should be monitored continuously and clinicians should expect that these infants might require a substantial increase in respiratory support or resuscitation. Thus, morphine should not be administered unless resuscitative equipment is available immediately, staff are trained appropriately, and both the risks and potential benefits are considered carefully. Difficulties in measuring infant pain are widely recognised, and the methodology used in our trial to measure both analgesic efficacy and side-effects of a pharmacological intervention sets new standards for the conduct of clinical trials of analgesics in infants.

We studied infants who required both a routine blood test and retinopathy of prematurity screening on the same morning. We aimed to test whether one dose of oral morphine sulphate (100 μg/kg) administered to non-ventilated premature infants before heel lancing and retinopathy of prematurity screening would provide analgesia, reduce physiological instability, and be safe.^13^ We chose an oral dose of 100 μg/kg (with an estimated peak effect at 1 h)^14^ based on extrapolation from guidance in the British National Formulary for children, local practice guidelines for neonatal eye surgery, and findings of a previous incomplete trial.^15^ We assessed multimodal pain measures: noxious-evoked brain activity, reflex activity, physiology, and behaviour. To provide insights into how both morphine and retinopathy of prematurity screening alter infant physiology, we monitored infants' heart rate, respiratory rate, blood pressure, and oxygen saturation for 24 h before and after the clinical procedure. We evaluated drug safety by assessing the incidence of hypotension requiring ionotropes and of apnoeic episodes requiring resuscitative non-invasive positive pressure ventilation (NIPPV), both potential adverse effects of morphine.^16^

## Methods

### Study design and participants

We undertook a single-centre, masked, randomised, placebo-controlled trial at the Newborn Care Unit at the John Radcliffe Hospital (Oxford University Hospitals NHS Trust, Oxford, UK). This trial was supported by the National Perinatal Epidemiology Unit Clinical Trials Unit (NPEU CTU).

Each infant was studied on a single test occasion. Infants were eligible for inclusion if they were born prematurely at less than 32 weeks' gestation or with a birthweight less than 1501 g (fulfilling UK retinopathy of prematurity guidelines),^17^ were both inpatients at the time of the study and aged 34–42 weeks' gestation, and required a heel lance and retinopathy of prematurity screening on the same test occasion (referred to hereafter as the clinical procedure). Exclusion criteria are provided in the appendix. All infants were assessed for eligibility by a senior clinician. We reassessed eligibility at randomisation, study commencement, and before administration of morphine or placebo.

We obtained written informed parental consent for all infants. Approval was obtained from the Medicines and Healthcare products Regulatory Agency (MHRA) and Northampton Research Ethics Committee (15/EM/0310). This trial conformed to the standards set by the Declaration of Helsinki.

### Randomisation and masking

We randomised infants to receive either morphine or placebo, using a web-based facility hosted by the NPEU CTU, with a minimisation algorithm to ensure approximate balance of key demographics between the groups (gestational age at birth, gestational age at time of randomisation, intrauterine growth restriction, time on ventilation, time since morphine last given, presence of a gastric tube, and history of surgery). Morphine sulphate (at a concentration of 200 μg/mL) and placebo solutions were indistinguishable by colour, odour, and flow, and were dispensed in 10 mL glass amber bottles with tamper-evident caps and a pack identification label (appendix). Researchers, clinicians, outcome assessors, and parents were masked to treatment allocation. In the event of an emergency, treatment allocation could be unmasked by a member of the clinical team using a single-use access code on the randomisation website. In the event of an infant becoming ineligible after randomisation, the study was postponed and recommenced when the infant became eligible, without rerandomisation.

### Procedures

A study timeline is provided in the appendix. Continuous electronic data capture of heart rate, respiratory rate, and oxygen saturation began approximately 24 h before the clinical procedure to establish a baseline of clinical stability for every infant. We recorded all changes in oxygen requirement, and measured blood pressure every 6 h.

Approximately 60 min before the clinical procedure, we gave infants one dose of either morphine sulphate (100 μg/kg) or placebo (of equivalent volume). We calculated the volume of the dose using the infant's working weight (the most recent weight in the infant's medical notes and used on their current drug prescription chart). We administered the dose orally (via syringe) or via a nasogastric tube (flushed with aspirate). Mydriatic eye drops (tropicamide 1% and phenylephrine 2·5%) were administered at 60 min and again at 45 min before the clinical procedure. Electroencephalography (EEG) and electromyography (EMG) electrodes were then sited, as described in the appendix.

Shortly before the clinical procedure, we swaddled the infant (to provide non-pharmacological pain relief), began video monitoring, and did a control heel lance (lancet was rotated and held against the foot, with the blade released into the air). This procedure was followed by the clinically required heel lance, approximately 60 min post administration of morphine or placebo. No non-essential or additional blood tests were done. After the heel lance and blood collection, we ensured that infants were fully settled and did not exhibit behavioural or physiological signs of distress before the retinopathy of prematurity examination. A senior opthalmologist performed all retinopathy of prematurity examinations. Topical local anaesthetic (proxymetacaine 0·5%) eye drops were instilled before insertion of an eyelid speculum, and binocular indirect ophthalmoscopic examination was completed using a Flynn style indenter.

After the clinical procedure, EEG and EMG leads were removed and physiological recordings and documentation of oxygen requirements continued for 24 h. A skilled neonatal nurse or paediatric doctor from the research team remained present for a minimum of 6 h after drug administration.

### Outcomes

The co-primary outcome measures were a behavioural pain score calculated after retinopathy of prematurity screening using the Premature Infant Pain Profile–Revised (PIPP-R),^18^ and the magnitude of noxious-evoked brain activity in response to heel lancing, measured using a validated EEG template (appendix).^11^ We calculated the PIPP-R score in the 30 s period after retinopathy of prematurity screening (after removal of the speculum following examination of the second eye). A PIPP-R score of 6 or lower indicates little or no pain and a score greater than 12 indicates moderate-to-severe pain.^19^

Secondary outcome measures were reflex withdrawal and the PIPP-R score after heel lancing (appendix). To assess the nociceptive specificity of outcome measures, we also assessed the PIPP-R score, magnitude of noxious-evoked brain activity, and reflex withdrawal for the control heel lance. Background brain activity and reflex withdrawal activity were also assessed in a baseline period during which the infant's foot was gently held but no stimuli were applied.

We assessed the clinical stability of infants by considering episodes of oxygen desaturation, bradycardia, tachycardia, and apnoea, and requirements for an increase in respiratory support, during the 6 h and 24 h periods after the clinical procedure, which were also secondary outcomes of the study. Episodes of oxygen desaturation were identified from the peripheral oxygen saturation signal as periods during which oxygen saturation fell below 80% for at least 10 s. Episodes of bradycardia were identified as periods during which the heart rate fell below 100 beats per min (bpm) for at least 15 s. Episodes of tachycardia were defined as periods during which the heart rate was greater than 200 bpm for at least 15 s (appendix). Apnoeic episodes were identified from clinical records or by retrospective review of the impedance pneumograph for breathing pauses longer than 20 s during bradycardic episodes (appendix). Increases in respiratory support were defined as a significant increase in oxygen requirement or an increase in respiratory support modality (appendix).

We assessed drug safety by calculating the incidence of apnoea requiring NIPPV and the incidence of hypotension requiring ionotropes in the 24 h period post administration of morphine or placebo. Clinicians on the research team recorded a description of adverse events that occurred in the 24 h period post administration of morphine or placebo, including action taken, severity, and causality of the event, identified by consultation of the clinical team and review of clinical records (appendix). All serious adverse events were reviewed by the Data Monitoring Committee.

### Statistical analysis

We calculated that a sample size of 132 infants would allow detection of a clinically meaningful reduction in pain scores (a clinically significant reduction in PIPP-R scores was defined as 2 points),^13^ from a conservative post-retinopathy of prematurity screening mean PIPP-R score of 8·3 (SD 3·5) from a previous study,^20^ with power of 90% (p<0·05; two-tailed). We considered a 40% reduction in noxious-evoked brain activity clinically meaningful, since a similar reduction in adults corresponds to significantly lower verbally reported pain scores.^21^ A sample size of 132 infants was also required for this co-primary outcome measure, with power of 90% (p<0·05; two-tailed). We inflated the sample size to 156 infants (78 per arm) to account for multiple births (assuming a 25% rate and an intra-cluster correlation coefficient of 0·5) and 10% loss to follow-up.

Trial outcomes were analysed and reported according to the trial protocol (version 6.0) and statistical analysis plan (version 2.0). Changes to the protocol (because of early trial cessation) are listed in the statistical analysis plan and the appendix. Analysis was per protocol, and a p value of 0·05 (two-sided 5% significance level) was deemed significant for all outcome measures.

We reported mean (SD) or median (IQR) values according to whether data were normally distributed or skewed. We calculated mean or median differences with 95% CIs and p values. We compared PIPP-R scores between groups using *t* tests. The magnitude of noxious-evoked brain activity and reflex withdrawal activity were compared between groups using a Wilcoxon rank-sum test, with the Hodges-Lehmann estimator used to calculate median differences with 95% CIs. We calculated SE of the median using bootstrap sampling. We assessed inter-rater and intra-rater reliability of PIPP-R scoring using intra-class correlation from a random-effects model with 95% CIs. Intra-rater reliability was 0·98 (95% CI 0·97–0·99) for heel lance PIPP-R scores and 0·97 (0·94–0·99) for retinopathy of prematurity screening. Inter-rater reliability was 0·98 (0·95–0·99) for heel lance and 0·89 (0·79–0·95) for retinopathy of prematurity screening.

For episodes of bradycardia, tachycardia, and oxygen desaturation, we standardised the difference in counts in the 6 h and 24 h periods before and after the clinical procedure, to adjust for the number of preprocedure episodes for every infant. We defined the standardised difference in number of episodes in the periods before and after the clinical procedure as the difference in number of episodes, as a proportion of the total number of episodes, for every infant, symmetrically in both the 6 h and 24 h periods (eg, in the 24 h period post procedure relative to the 24 h period preprocedure). To avoid issues caused by zero counts, we added a negligible constant term (0·01) to each count before standardisation. We compared standardised differences between treatment groups using a Wilcoxon rank-sum test, with the Hodges-Lehmann estimator used to calculate median differences with 95% CIs. Infants with new-onset apnoea or an increased number of apnoeic episodes after the clinical procedure were compared using risk ratios (RRs). The number of infants requiring increased respiratory support after the clinical procedure was compared using risk differences (although we planned to use RR analysis according to the statistical analysis plan, this could not be done because no infants in the placebo group needed increased respiratory support). In a post-hoc analysis, we calculated the average time courses of physiological variables (heart rate, respiratory rate, and oxygen saturation) over the 48 h trial period and compared them between groups using non-parametric cluster analysis (appendix). We analysed data with Stata SE (version 13.1) and MATLAB (R2017a), and East (version 6.4) was used for the stopping boundary and safety analysis.

A trial stopping boundary was predefined based on the event rate of apnoeic episodes requiring resuscitative NIPPV (bag valve mask [visionary single patient use manual resuscitator; Marshall Airway Products, Radstock, UK] or Neopuff [Fisher & Paykel Healthcare, Auckland, New Zealand]). The boundary was chosen by the Data Monitoring Committee before any analyses, after review of hypothetical trial scenarios and defined using a group sequential method with a one-sided gamma spending function (γ=4·5, type I error rate=0·2, estimated power=0·79). The selected boundary was based on a control group event rate of 7% and a difference between the group event rates of 12%. After 25 infants were randomised and studied, the Data Monitoring Committee convened for a safety review as planned and were provided with the stopping boundary graph, clinical stability and safety data summarised by arm, and detailed summaries of safety and adverse events, to consider evidence for benefit and harms.

This trial is registered with the European Clinical Trials Database (number 2014-003237-25).

### Role of the funding source

The funder had no role in study design, data collection, data analysis, data interpretation, or writing of the report. The corresponding author had full access to all data in the study and had final responsibility for the decision to submit for publication.

## Results

**Figure 1:**
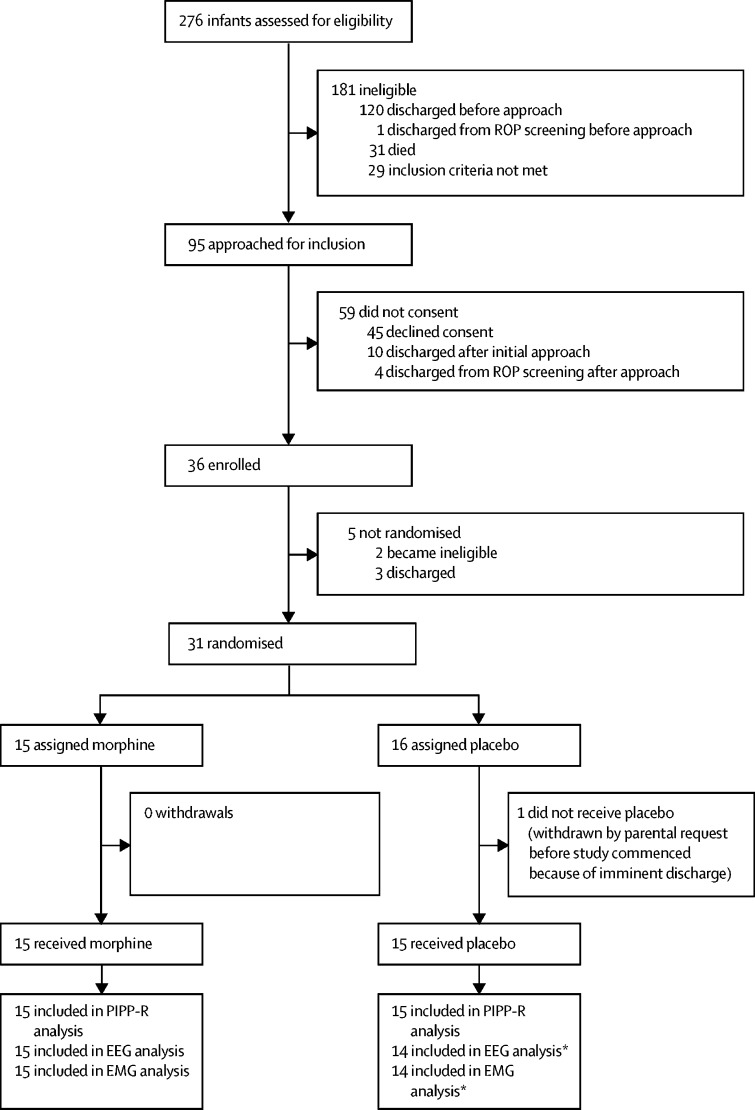
Trial profile EEG=electroencephalography. EMG=electromyography. PIPP-R=Premature Infant Pain Profile–Revised. ROP=retinopathy of prematurity. *One excluded because of artifact.

Between Oct 30, 2016, and Nov 17, 2017, 31 infants aged 34–39 weeks' gestation (at the time of the study), who required retinopathy of prematurity screening and heel lancing on the same occasion, were enrolled and randomly assigned either 100 μg/kg of oral morphine (n=15) or placebo (n=16); one infant assigned placebo was withdrawn from the study before monitoring began (figure 1).

The Data Monitoring Committee reviewed safety data after recruitment of 25 infants (on Dec 4, 2017), and the predefined stopping boundary had been crossed. The Data Monitoring Committee requested data for all 31 infants who had been randomised. They concluded there was sufficient evidence to suggest that oral morphine at the dose used in our study has the potential to cause harm without any suggestion of analgesic benefit. After considering the evidence and wider implications, the Trial Steering Committee concluded that the trial should not continue in its present form and recommended that the investigators review the data to guide further investigation. The trial was stopped by the Central Monitoring Team on March 15, 2018 (appendix).

**Figure 2:**
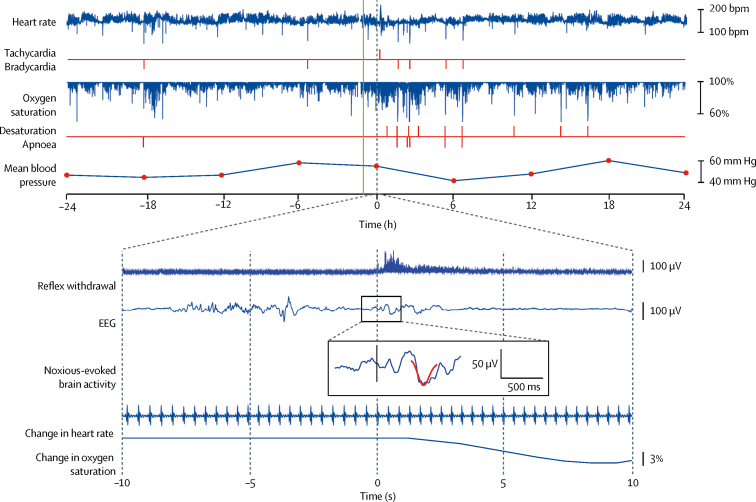
Example of data recorded in an infant assigned morphine 48 h records of heart rate, oxygen saturation, and mean blood pressure every 6 h are shown for the 24 h periods before and after the clinical procedure. Episodes of tachycardia, bradycardia, oxygen desaturation, and apnoea are identified (red vertical lines). The reflex withdrawal activity, EEG activity, and change in heart rate and oxygen saturation are shown in the 10 s before and after the heel lance. The noxious-evoked brain activity template is projected onto the EEG trace (overlaid in red). The time of drug administration is indicated by the green vertical line (approximately 1 h before the clinical procedure). This infant was chosen as a representative example because he had all clinical stability events (tachycardia, bradycardia, oxygen desaturation, and apnoea) and had clear changes in reflex withdrawal, brain activity, and physiology to the heel lance. Traces of noxious-evoked brain activity to the heel lance and 48 h physiological traces for all individual infants are in the appendix. bpm=beats per min. EEG=electroencephalogram.

There were no deviations from exclusion or inclusion criteria. Infant demographics and clinical characteristics at birth and at the time of intervention are reported in table 1 according to group allocation. Most baseline characteristics were well balanced between treatment groups; however, a few variables were less well balanced (eg, the number of infants who required ventilatory support), which is expected in view of the small sample size. Figure 2 shows example data for one infant for each recording modality.

**Table 1:** Infant demographics

		**Morphine (n=15)**	**Placebo (n=15)**
**Characteristics at birth**			
Gestational age (weeks)*†		28·1 (26·3–30·1)	28·6 (27·9–29·7)
Birthweight (g)		1107 (329)	1173 (350)
Birthweight Z-score		−0·4 (0·9)	−0·2 (1·0)
Intrauterine growth restriction*		2 (13%)	3 (20%)
Apgar score at 10 min‡		10·0 (9·0–10·0)	10·0 (8·0–10·0)
Mode of delivery			
	Spontaneous vaginal delivery	8 (53%)	5 (33%)
	Caesarean section	7 (47%)	10 (67%)
Male sex		12 (80%)	8 (53%)
Female sex		3 (20%)	7 (47%)
Multiple pregnancy		4 (27%)	4 (27%)
**Characteristics at time of randomisation**			
Gestational age (weeks)*†		34·7 (34·1–35·1)	34·7 (34·1–35·1)
Time on ventilation (days)*§		8·0 (1·0–20·0)	3·5 (2·0–19·5)
Time since morphine last given (days)*¶		46·5 (33·5–49·0)	19·0 (15·0–39·0)
Presence of gastric tube*		14 (93%)	15 (100%)
Intraventricular haemorrhage (grade I or II)		3 (20%)	2 (13%)
History of surgery*		0 (0%)	1 (7%)
**Characteristics at time of clinical procedure**			
Gestational age (weeks)†		35·0 (34·3–35·4)	34·9 (34·3–36·3)
Postnatal age (days)		50 (28–58)	49 (43–59)
Weight (g)		2049 (426)	2127 (331)
Duration of ROP screening (s)		97 (82–108)	91 (83–110)
Diagnosis of ROP		2 (13%)	2 (13%)
Level of care			
	Intensive care unit	1 (7%)	1 (7%)
	High-dependency unit	5 (33%)	9 (60%)
	Low-dependency unit	9 (60%)	5 (33%)
Respiratory support modality			
	Self-ventilating	9 (60%)	8 (53%)
	Low-flow oxygen therapy	2 (13%)	1 (7%)
	High-flow oxygen therapy	4 (27%)	6 (40%)
Time between IMP administration and heel lance (min)		61 (57–66)	63 (58–70)

Data are median (IQR), mean (SD), or number (%). IMP=investigational medicinal product. ROP=retinopathy of prematurity.

* Criteria used in minimisation algorithm for randomisation.

† Postmenstrual age is often used in neonatal practice. In our unit, the infants' gestational age is recorded each day in the medical and nursing notes; therefore, we have used this nomenclature.

‡ Data are for 15 infants in the morphine group and 13 in the placebo group.

§ Data are for six infants in the morphine group and 12 in the placebo group.

¶ Data are for four infants in the morphine group who previously received morphine and seven in the placebo group.

**Figure 3:**
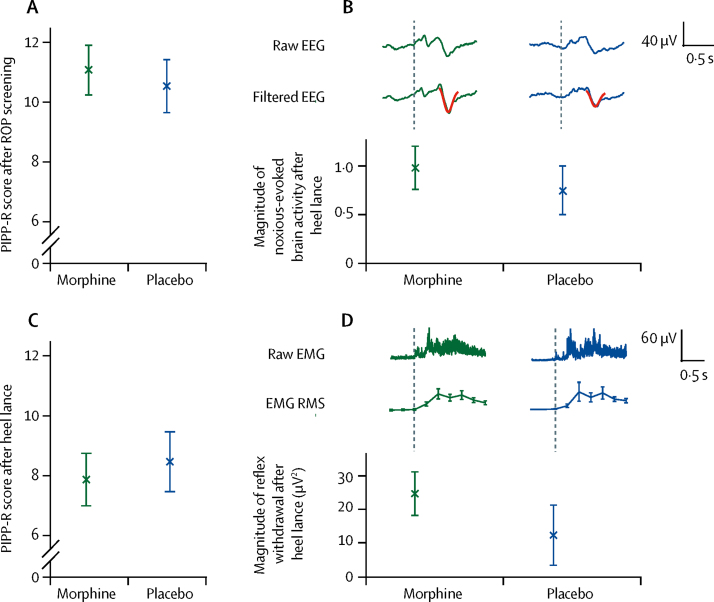
Co-primary and secondary outcome measures of analgesic efficacy Co-primary outcomes are shown in (A) and (B). (A) Mean (SE) PIPP-R scores after ROP screening. (B) Median (SE) magnitude of noxious-evoked brain activity after heel lance. The (Woody) filtered EEG is shown overlaid with the template of noxious-evoked brain activity (in red). Secondary outcomes are shown in (C) and (D). (C) Mean (SE) PIPP-R score after heel lance. (D) Median (SE) magnitude of reflex withdrawal activity after heel lance. Magnitude is quantified using RMS in 250 ms windows. EEG=electroencephalogram. EMG=electromyography. PIPP-R=Premature Infant Pain Profile-Revised. RMS=root-mean-square. ROP=retinopathy of prematurity.

The co-primary outcome measures used to assess morphine analgesic efficacy were PIPP-R score after retinopathy of prematurity screening and magnitude of noxious-evoked brain activity after heel lancing. PIPP-R scores after retinopathy of prematurity screening did not differ significantly between infants assigned morphine (mean 11·1 [SD 3·2]) and those allocated placebo (10·5 [3·4]; mean difference 0·5, 95% CI −2·0 to 3·0; p=0·66; figure 3). Similarly, the magnitude of noxious-evoked brain activity after heel lancing did not differ significantly between infants assigned morphine (median 0·99 [IQR 0·40–1·56]) and those allocated placebo (0·75 [0·33–1·22]; median difference 0·25, 95% CI −0·16 to 0·80; p=0·25; figure 3). EEG responses for all individual infants are shown in the appendix. The secondary outcome measures of PIPP-R score after the heel lance and the magnitude of reflex withdrawal activity evoked by the heel lance did not differ between the two groups. Mean PIPP-R score after heel lance was 7·9 (SD 3·4) with morphine and 8·5 (3·9) with placebo (mean difference −0·6, 95% CI −3·3 to 2·1; p=0·66). The magnitude of reflex withdrawal was median 24·8 (IQR 19·7–44·8) with morphine and 12·4 (6·1–46·3) with placebo (median difference 8·9, 95% CI −12·0 to 22·4; p=0·48; figure 3). The magnitude of each pain-related outcome measure increased significantly after the clinical procedure compared with control stimuli and non-noxious background activity (appendix), showing that the measures were discriminative and appropriate for assessing analgesic efficacy in this population.

**Figure 4:**
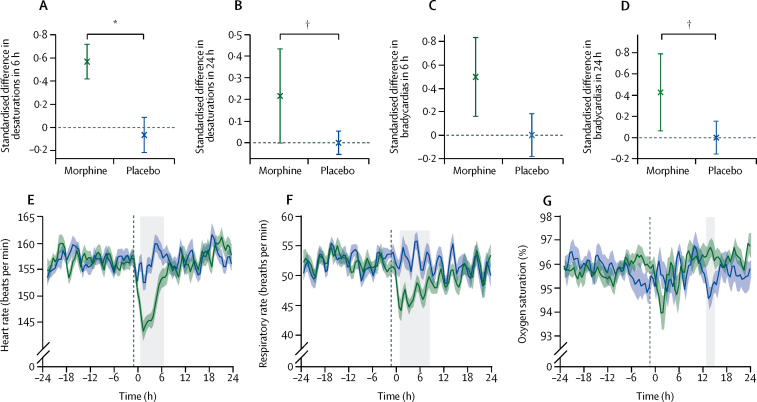
Assessments of physiological stability (A) Median (SE) of the standardised difference in number of episodes of desaturation in the 6 h period after the clinical procedure compared with the 6 h period before. (B) Median (SE) of the standardised difference in number of episodes of desaturation in the 24 h period after the clinical procedure compared with the 24 h period before. (C) Median (SE) of the standardised difference in number of episodes of bradycardia in the 6 h period after the clinical procedure compared with the 6 h period before. (D) Median (SE) of the standardised difference in number of episodes of bradycardia in the 24 h period after the clinical procedure compared with the 24 h period before. (E) Mean (SE) heart rate during the 48 h monitoring period. (F) Mean (SE) respiratory rate during the 48 h monitoring period. (G) Mean (SE) oxygen saturation during the 48 h monitoring period. (E–G) Individual infant traces are baseline-corrected to the average baseline across all infants. Time zero is the point of the clinical procedure. Black vertical dashed line indicates the time of administration of morphine or placebo. Grey boxes indicate periods during which the treatment groups differed significantly. *p=0·0007. †p=0·019.

The clinical stability of each infant was assessed over 48 h (24 h before and after the clinical procedure). Infants assigned morphine had significantly more episodes of oxygen desaturation in the 6 h and 24 h periods after the clinical procedure, and significantly more episodes of bradycardia in the 24 h period after the clinical procedure, compared with those allocated placebo (table 2; figure 4). There were no differences in the number of episodes of tachycardia.

**Table 2:** Clinical stability

	**Morphine**	**Placebo**	**Median difference (95% CI)**	**p value**
**6 h**				
Oxygen desaturation	0·57 (0·00 to 0·99)	−0·06 (−0·65 to 0·00)	0·66 (0·36 to 1·00)	0·0007
Bradycardia	0·50 (0·00 to 0·99)	0·00 (0·00 to 0·98)	0·33 (0·00 to 0·98)	0·07
Tachycardia	0·00 (0·00 to 0·00)	0·00 (0·00 to 0·00)	0·00 (−0·16 to 0·00)	0·32
**24 h**				
Oxygen desaturation	0·22 (−0·02 to 0·98)	0·00 (−0·25 to 0·08)	0·33 (0·03 to 0·75)	0·019
Bradycardia	0·43 (0·00 to 1·00)	0·00 (−0·50 to 0·60)	0·43 (0·00 to 1·00)	0·019
Tachycardia	0·00 (−0·50 to 0·98)	0·00 (0·00 to 0·00)	0·00 (−0·38 to 0·98)	0·57

Data are median (IQR) of the standardised difference in number of episodes before and after intervention, unless otherwise stated.

Eight (53%) of 15 infants who received morphine developed new-onset apnoea or an increase in the number of apnoeic episodes in the 24 h period after the clinical procedure, compared with three (20%) of 15 infants who received placebo (RR 2·7, 95% CI 0·9–8·2; p=0·085). More infants assigned morphine had new-onset or increased apnoea in the 6 h period after the clinical procedure compared with those allocated placebo (seven of 15 infants *vs* three of 15 infants), although the difference was not significant (RR 2·3, 95% CI 0·7–7·4; p=0·15). Significantly more infants allocated morphine required an increase in respiratory support in the 6 h period after the clinical procedure compared with those assigned placebo (four of 15 infants *vs* none of 15 infants; risk difference 0·3, 95% CI 0·0–0·5; p=0·020), and in the 24 h period after the clinical procedure (five of 15 infants *vs* none of 15 infants; risk difference 0·3, 95% CI 0·1–0·6; p=0·006).

Drug safety was assessed by considering the number of infants who had apnoeic episodes requiring resuscitation with NIPPV and the incidence of hypotension requiring treatment with inotropes in the 24 h period after drug administration. The predefined safety stopping boundary was passed, because three (20%) of 15 infants assigned morphine had apnoeic episodes requiring resuscitation with NIPPV in the 24 h period after drug administration, compared with no infants assigned placebo (difference in proportion 0·2, 80% CI [adjusted to allow for planned multiple analyses] 0·05–1·00; p=0·085; significant at the 20% level allowing for the prespecified stopping boundary). No infant needed inotropes. Blood pressure was similar in infants in both treatment groups (appendix).

11 (37%) of the 30 infants had adverse events, two of whom had unforeseeable serious adverse events (table 3). Eight (53%) of the 15 infants who received morphine had respiratory adverse events attributed as possibly or probably related to drug administration. Of the 15 infants who received placebo, one had a mild respiratory adverse event. Two infants in each treatment group were diagnosed with retinopathy of prematurity, an expected foreseeable serious adverse event.

**Table 3:** Adverse events

	**Onset of event post drug (h, min)**	**Treatment**	**Grade**	**Attribution**	**Allocation**
**Adverse events**					
Nasal congestion	11 h, 56 min	Saline drops	Mild	Not related	Placebo
Rash	4 h, 4 min	Cream	Mild	Not related	Placebo
Profound desaturation episodes	17 h, 59 min	Facial oxygen	Mild	Not related	Placebo
Recurrent desaturation episodes	8 h, 9 min	Stimulation	Mild	Possibly	Morphine
Recurrent desaturation episodes	1 h, 58 min	Facial oxygen	Mild	Possibly	Morphine
Apnoea	2 h, 13 min	NIPPV; increase high-flow oxygen	Moderate	Possibly	Morphine
Recurrent apnoeic episodes	2 h, 39 min	Stimulation; increase low-flow oxygen	Moderate	Possibly	Morphine
Recurrent apnoeic episodes	1 h, 28 min	Stimulation (× 3); NIPPV (× 3)	Moderate	Possibly	Morphine
Recurrent desaturation, bradycardia, and apnoeic episodes	2 h, 3 min	Commenced high-flow oxygen; feed volume reduction	Moderate	Possibly	Morphine
**Serious adverse events**					
Persistent hypoventilation and desaturation	6 h, 0 min	Moved to high-dependency unit; commenced high-flow oxygen	Moderate	Possibly	Morphine
Recurrent apnoeic episodes	6 h, 24 min	Unmasked by clinical team; moved to high-dependency unit; commenced high-flow oxygen; naloxone (× 2)	Moderate	Probably	Morphine

Adverse events are shown that occurred during the 24 h period post administration of morphine or placebo. NIPPV=non-invasive positive pressure ventilation.

Post-hoc exploratory analyses showed that administration of morphine resulted in a significant reduction in heart rate and respiratory rate compared with placebo. Morphine administration reduced the group average heart rate by a maximum of 13·9 bpm at 1·5 h after the clinical procedure (ie, approximately 2·5 h after drug administration), and heart rate was significantly lower in infants allocated morphine for 6 h (from 0·5 h to 6·5 h after the clinical procedure; p=0·0001; figure 4). The respiratory rate dropped by an average of eight breaths per min at 1·5 h after the clinical procedure, and it was significantly lower in the morphine group for 7·5 h (from 1 h to 8·5 h after the clinical procedure; p=0·003; figure 4). Similarly, 1·5 h after the clinical procedure, oxygen saturation dropped by an average of 2% in infants allocated morphine, although this reduction was not significantly lower than in infants assigned placebo (figure 4). More than 12 h after the clinical procedure, oxygen saturation of infants allocated placebo dropped by 1·2%, and this value was significantly different to that for infants assigned morphine for 2 h (from 13 h to 15 h after the clinical procedure; p=0·022; figure 4). The appendix includes 48 h oxygen saturation and heart rate traces for all infants.

## Discussion

This trial shows that administration of 100 μg/kg oral morphine before acutely painful clinical procedures in infants born prematurely (and aged 34–39 weeks' gestation at study) has a profound negative effect on respiratory stability, without any suggestion of analgesic efficacy (video). A multimodal approach was used to assess analgesic efficacy and safety, providing detailed evidence of the effects of morphine on infant nociceptive and physiological activity. Behavioural pain scores, noxious-evoked brain activity, and reflex withdrawal activity did not differ between morphine and placebo groups. The study was underpowered for the co-primary outcome measures, because of early trial cessation, and we therefore cannot conclude whether morphine provided effective analgesia at this dose. However, a trend was noted across modalities that infants who received morphine had greater noxious-evoked activity, suggesting that even if the trial had continued to completion, we would have been unlikely to observe an analgesic effect of morphine.

This trial suggests that oral morphine at a dose of 100 μg/kg in non-ventilated infants has the potential for harm. The profound respiratory effects observed justified trial cessation and lead us to recommend that oral morphine (at this dose) should not be given to non-ventilated premature infants for acute pain relief during retinopathy of prematurity screening. The age range in this study was restricted to infants requiring retinopathy of prematurity screening at 34–42 weeks' gestation, so we cannot ascertain the effects of oral morphine in younger or older infants. However, international paediatric formularies—eg, the British National Formulary for children—recommend an oral dose of 50–100 μg/kg every 4 h in infants aged 1–2 months, and 50 μg/kg of intravenous morphine every 6 h to treat pain in neonates, which is roughly equivalent to the 100 μg/kg oral dose administered in this study, assuming an oral bioavailability of approximately 50%.^22^ Our data suggest that in non-ventilated premature infants of 1–2 months' postnatal age, these doses could cause substantial respiratory adverse effects, requiring resuscitative respiratory support or a change in respiratory support modality. This effect might be due to immature glucuronidation and reduced clearance of morphine metabolites, because these processes are not mature in the first 2 months of life.^23^

Although an intravenous morphine dose of 10–30 μg/kg provided effective analgesia in infants receiving continuous positive airway pressure in a previous study,^24^ severe apnoeic episodes requiring substantial intervention were reported in 9% of participants (who were very premature), and consistent with the findings of our study, a reduction in heart rate and respiratory rate was suggested. Similarly, a dose of 100 μg/kg of intravenous morphine in ventilated premature infants provided effective pain relief for central line placement in a previous study,^25^ but with significantly increased ventilation requirements compared with infants who received tetracaine. Findings of another retrospective study^16^ showed that five of 43 infants who received approximately 50–100 μg/kg of intravenous morphine for central line placement had respiratory depression requiring intervention or increased respiratory support, compared with none in a control group.

It is possible that if we had lowered the dose of oral morphine that the adverse respiratory outcomes could have been reduced. However, it seems unlikely that a lower dose would have provided effective analgesia. Although our national drug formulary recommends 50–100 μg/kg of morphine orally for pain, neonatal drug guidelines from other countries (eg, Australia) recommend higher oral doses of 100–200 μg/kg for pain in neonates.^26^ Our local practice guidelines also recommend that an oral dose of 100 μg/kg is given to infants requiring laser eye surgery. In a pilot trial that was started but not completed,^15^ six non-ventilated infants were administered a much larger oral dose of morphine (200 μg/kg) for retinopathy of prematurity screening. These researchers did not report any adverse effects. We therefore determined that, on balance, 100 μg/kg was a justifiable dose.

The acceptable balance of benefit and harm for any treatment is contextually dependent. All infants in our trial were clinically stable before the study started, and most infants who received morphine were cared for in a low-dependency setting and self-ventilating in air. Although ventilatory support can be routinely and expertly provided in neonatal care, escalation of oxygen therapy or level of care can be costly, result in prolongation of admission and distress to parents, and be viewed as a considerable setback. Ideally, morphine should be titrated to provide patients with optimum analgesic benefit and minimum adverse effects. The likely requirement for increased respiratory support would need to be expected, manageable, and justified by analgesic benefits. In our study, we noted considerable adverse effects before any suggestion of benefit, suggesting a non-existent therapeutic window in this context. Doses of morphine that are routinely administered intravenously to ventilated infants^27^ might provide effective analgesia in premature infants, albeit limited analgesic efficacy has been reported.^3^ Because respiratory adverse effects can be managed well in ventilated infants, the benefit of morphine administration for sedation might outweigh the risks, and results from this study cannot be interpreted to suggest that ventilated infants should not be given morphine. Controversy remains over the analgesic efficacy of intravenous morphine for procedural pain in ventilated infants,^3^ and comprehensive assessment of pain-related brain activity could help settle this debate.

The single bolus dose of morphine administered in our trial produced clinically significant cardiorespiratory effects; the heart rate and respiratory rate were significantly lower in infants assigned morphine for 6–8 h after the clinical procedure. This finding corresponds with the half-life of morphine in premature infants.^28^ A comparable degree of cardiorespiratory depression has been seen in ventilated infants receiving intravenous morphine infusions.^29^ This finding highlights the importance of comprehensively evaluating the side-effects of pain-relieving drugs through detailed physiological recordings and clinical observations.

Due to early trial cessation, our study was underpowered to detect significant effects in the co-primary outcome measures. Although mydriatic eye drops were given at approximately the same time as the drug, which might delay gastric emptying,^30^ this does not seem to have prevented morphine absorption. A limitation of our study design was that timing of peak analgesic efficacy of oral morphine is unknown; however, the time courses of cardiac and respiratory effects suggest a drug effect at the time of the clinical procedure. It is unlikely that an absence of analgesic efficacy is related to a lack of absorption or poor timing of drug administration.

In view of the challenges in measuring analgesic efficacy in non-verbal infants,^31^ the recorded increases in the measures of noxious-evoked brain activity, reflex withdrawal, and PIPP-R scores in response to the clinical procedure confirm the suitability of these approaches to assess analgesic efficacy. The multimodal approach used in our trial to assess both analgesic efficacy and drug safety can provide detailed mechanistic insight into the precise time course of physiological effects of potential analgesics. Although this multimodal methodology cannot be implemented easily into standard clinical practice, this work highlights the importance and feasibility of using this approach in future clinical trials of analgesics in infants.^31^

Our trial provides further evidence that retinopathy of prematurity screening is a painful and destabilising procedure. In a meta-analysis of pain relief for retinopathy of prematurity screening,^32^ with topical anaesthetic the median PIPP score was 15, reducing to 11 with the addition of sweet taste. In our placebo group, the mean PIPP-R score was 10·5, and 20% of infants developed new-onset or increased apnoeic episodes in the 24 h after the clinical procedure. This finding corresponds with previous work^7^, ^8^ and provides further evidence that pain during retinopathy of prematurity screening is not being managed adequately in clinical practice. Post-procedure monitoring requirements should be considered carefully, particularly in infants who require retinopathy of prematurity screening after discharge in an outpatient setting. Novel imaging techniques for retinopathy of prematurity screening are being developed but are currently used as an adjunct to indirect ophthalmoscopy rather than as a replacement.^33^ Further clinical trials should be done to identify effective analgesia for this procedure.

In conclusion, oral morphine at a dose of 100 μg/kg has the potential for harm with no suggestion that it provides analgesic efficacy for acute clinical procedures in non-ventilated infants born prematurely (and aged 34–39 weeks' gestation at study). However, because of early trial cessation, we cannot draw conclusions about the analgesic efficacy of oral morphine; obtaining a large enough sample size to test this objective would have required exposing infants to an unacceptable risk of respiratory adverse events. We do not recommend this dose of morphine for use as pain relief during binocular indirect ophthalmoscopy retinopathy of prematurity screening. Using multimodal outcome measures, along with detailed physiological recordings, provides a rigorous approach to assess analgesic efficacy and adverse effects, leading to a greater mechanistic understanding of the effects of a drug, and is desirable in future clinical trials of analgesics in infants.

For the **trial protocol and statistical analysis plan** see https://www.npeu.ox.ac.uk/poppi/protocol-publications

## Supplementary Materials

Supplementary appendix

Supplementary video A summary of a clinical trial investigating the analgesic efficacy and safety of oral morphine in premature infants.
