# Supplementary material for: Analgesic efficacy and safety of morphine in the Procedural Pain in Premature Infants (Poppi) study: randomised placebo-controlled trial
Source: Lancet. 2018 Dec 15;392(10164):2595–605. doi: 10.1016/S0140-6736(18)31813-0 (PMC6294828; doi:10.1016/S0140-6736(18)31813-0)
Supplement: Supplementary appendix [file mmc1.pdf]

THE LANCET

Supplementary appendix

This appendix formed part of the original submission and has been peer reviewed. We post it as supplied by the authors.

Supplement to: Hartley C, Moultrie F, Hoskin A, et al. Analgesic efficacy and safety of morphine in the Procedural Pain in Premature Infants (Poppi) study: randomised placebo-controlled trial. *Lancet* 2018; published online Nov 30. http://dx.doi.org/10.1016/S0140-6736(18)31813-0.

Supplementary Methods

Exclusion criteria

Infants were not eligible for inclusion if they had grade 3 or 4 intraventricular haemorrhages, had short bowel syndrome, were nil by mouth, had congenital malformations or genetic conditions known to affect neurodevelopment, had received analgesics or sedatives in the previous 24 hours or opiates in the previous 72 hours, had a documented opiate sensitivity, or were born to mothers who regularly used opiates during pregnancy or while breastfeeding or expressing breast milk. No infants were mechanically ventilated at the time of study.

Drug manufacture

Morphine sulphate (at a concentration of 200μg/ml) and placebo solutions were manufactured by Stockport Pharmaceuticals Production Unit in accordance with Good Manufacturing Practice (GMP), delivered to the pharmacy at the John Radcliffe Hospital in 10ml glass amber bottles with tamper-evident caps and a pack ID label, and dispensed to the Newborn Care Unit. The solutions were indistinguishable by colour, odour and flow.

Experimental recording techniques

Continuous heart rate from ECG, respiratory rate from an impedance pneumograph, and oxygen saturations from a pulse oximeter were recorded at a sampling rate of 1Hz using an IntelliVue MX800 Phillips monitor and downloaded continuously using ixTrend software (iexcellence). ECG and the impedance pneumograph were recorded at sampling rates of 250Hz and 62.5Hz respectively. Key trial and clinical events were electronically annotated. Episodes of desaturation, bradycardia and tachycardia were identified from the electronic recordings of the oxygen saturation and ECG. If more than one event happened within 60 seconds, it was recorded as a single event. Periods of interrupted signal, indicating movement artifacts or loss of signal, were excluded from the analysis. If the heart rate from the ECG was interrupted, then the pulse rate from the pulse oximeter was used to identify episodes of bradycardia and tachycardia. Blood pressure was non-invasively measured approximately 6-hourly using a standard neonatal cuff and recorded on the vital signs monitor and a paper cotside log. If the downloading of the physiological recordings were interrupted due to technical error, the episodes of desaturation, bradycardia, tachycardia and apnoea were recorded from clinical notes or retrospective review of the monitor log.

Facial expressions were filmed throughout the clinical procedure; a clear view of the face was recorded for 15 seconds before and 30 seconds after the heel lance control, the heel lance and the ROP screening. The PIPP-R score following the ROP screening was calculated in the 30-second period after removal of the speculum from the second eye.

The duration of ROP screening was determined in each infant retrospectively from the video recordings, as the time from first insertion of the speculum into the first eye to removal of the speculum from the second eye.

Electrophysiological activity was acquired from DC - 400 Hz with a sampling rate of 2 kHz using the SynAmps RT 64-channel headbox and amplifiers (Compumedics Neuroscan), and recorded using CURRYscan7 neuroimaging suite (Compumedics Neuroscan). Eight EEG recording electrodes (Ambu Neuroline disposable Ag/AgCl cup electrodes) were positioned on the scalp at Cz, CPz, C3, C4, FCz, T3, T4 and Oz, according to the modified international 10-20 System, with reference and ground electrodes at Fz and the forehead respectively. EEG conductive paste (Elefix EEG paste, Nihon Kodhen) was used to optimise contact and impedances were maintained at <5 kΩ with EEG preparation gel (Nuprep gel, D.O Weaver and Co.). Bipolar EMG electrodes (Ambu Neuroline 700 solid gel surface electrodes) were sited on the biceps femoris of each leg to measure reflex withdrawal. The electrodes were positioned on the infants after drug administration, and approximately 30 minutes before the clinical procedures. Infants were not distressed by the electrode placement.

The heel lance and heel lance control were time-locked to the electrophysiological recordings using an event-detection interface[1], and to the video recording using an LED light activated by the experimenter.

Clinical Procedures

Heel lances were performed as part of the infant's routine care. Heel lances were performed on the medial or lateral plantar surface of the heel. In older infants a BD Microtainer Quikheel Infant Lancet (Becton, Dickinson and Company, New Jersey, USA) with a penetration depth of 1.0 mm was used, and in younger infants, a BD Quikheel Preemie Lancet with a penetration depth of 0.85 mm was used, as per local clinical practice. Following the heel lance the foot was not squeezed for 30 seconds to ensure that the responses recorded were related to the noxious input. Prior to the heel lances, control heel lances were performed; the lancet was rotated by 90 degrees so that when the blade was released it did not penetrate the skin.

All infants required a heel lance and ROP screening on the same test occasion. Clinical procedures are often clustered in neonatal practice in order to provide protected rest periods when infants are undisturbed. In our unit, infants commonly have routine blood tests followed by ROP screening on the same morning. No unnecessary or additional blood tests were performed for the study. Infants were fully settled between the heel lance and ROP screening, and we did not begin the ROP screening if the infants displayed behavioural or physiological signs of distress.

Respiratory Support

Increased respiratory support was defined as a significant increase in oxygen requirement or an increase in ‘respiratory support modality’. ‘Respiratory support’ modality was classified on a graded scale from 1–4, according to the following definitions.

Grade 1:	self-ventilating in air
Grade 2:	low flow (0.01–0.35 litres/minute; 100% oxygen)
Grade 3:	high flow (1–8 litres/minute) or continuous positive airway pressure (CPAP) or duoPAP (21–100% oxygen)
Grade 4:	ventilator (21–100% oxygen)

A significant increase in oxygen requirement was defined as an increase in oxygen supply by more than 10%, a flow rate change of more than 1 litre/minute (if receiving high flow therapy) or a flow rate change of more than 0.04 litres/minute (if receiving low flow oxygen).

Outcome measures

PIPP-R scoring

The clinical pain scores were calculated using the validated Premature Infant Pain Profile ─ Revised (PIPP-R), which includes behavioural (facial expression changes) and physiological (heart rate and oxygen saturation changes) indicators, each rated on a 0─3 scale based on duration or degree of change. Facial expressions were scored offline by a trained investigator, and changes in heart rate and oxygen saturation were calculated from physiological recordings. Scores for gestational age and behavioural state were included if the physiological or behavioural variables scored a non-zero value[2].

EEG

Noxious-evoked brain activity was identified using a validated EEG-based template of noxious-evoked brain activity[3]. EEG activity was filtered from 0.5─70 Hz with a notch filter at 50Hz, epoched from 0.5 seconds pre-stimulus to 1 second post-stimulus, and baseline corrected to the pre-stimulus mean. EEG traces were Woody filtered, with a maximum jitter of ± 50ms, in the region of 400─700 ms after the stimulus, by identifying the maximum correlation with the template. The template was then projected onto each individual trial using singular value decomposition to calculate the magnitude of the noxious-evoked brain activity in the region 400─700 ms after the stimulus at the Cz electrode. The EEG response from 1 infant (who received placebo) was excluded from further analyses due to artefact.

EMG

Reflex withdrawal response to heel lancing and the control heel lance was measured using EMG. Data were filtered (10─500 Hz, notch filter at 50Hz and harmonics), baseline corrected to the pre-stimulus mean, rectified, and the root mean square (RMS) of the signal calculated in 250ms windows. The magnitude of the reflex withdrawal was calculated as the mean RMS activity in the 1000ms post-stimulus[4]. EMG from 1 infant (who received placebo) was excluded from further analyses due to artefact.

Physiological time courses

Average time courses were calculated for each physiological variable (heart rate, respiratory rate and oxygen saturation) by calculating the mean values for each infant in one-hour moving intervals, overlapping by half an hour, plotted at the end of each hour. The time course for each infant was baseline corrected by subtracting the mean in the first 23 hours of the recording (i.e. up until approximately the time of drug administration). Time courses between the morphine and placebo groups were compared using non-parametric cluster analysis as described by Maris and Oostenveld[5]. Briefly, time periods with differences between the morphine and placebo groups were identified by calculating a t-statistic at each time point. Significant differences in the time courses were defined to have occurred when the t-statistic was greater than the 97.5th percentile of the t-distribution. Clusters of significant activity were identified based on temporal adjacency of above threshold points. A cluster-

based test statistic was calculated by comparing the duration of the clusters with clusters identified from 10,000 random permutations of the data. For graphical representation in Figure 4, the traces were corrected by adding the mean baseline across all infants to better visualise the data.

Adverse events

A dedicated clinical member of the research team who was either a neonatal intensive care nurse or a paediatric doctor monitored each infant for a minimum of 6 hours following administration of the morphine or placebo. In the subsequent 18 hours, the nurse responsible for the clinical care of the infant was also responsible for trial monitoring. They measured the 6-hourly blood pressures, documented changes in oxygen requirement, recorded any apnoeas requiring intervention and recorded any adverse events. As this was a single centre trial, nurses were very familiar with the study protocol. Clinical members of the research team were also on-call throughout the 48-hour study period. Following this period two paediatric doctors who were members of the research team also reviewed the clinical notes of the infant to determine any adverse events (AEs) that occurred following administration of morphine or placebo. AEs were defined as any untoward medical occurrence in a participant. Serious adverse events (SAEs) were identified as medical occurrences that resulted in death, persistent or significant disability/incapacity, or prolongation of hospitalisation, or which were considered life-threatening, jeopardised the participant, or required intervention to prevent one of these consequences. Attribution and expectedness was determined by a clinical investigator masked to treatment allocation, according to the summary of Product Characteristics for Oramorph Oral Solution. SAEs were categorised as unrelated, possibly, probably or definitely related to the administration of trial medication, and graded as mild, moderate or severe. All SAEs labelled possibly, probably or definitely related, were considered related to the trial drug. Expected adverse events were also recorded, including suspected sepsis, anaemia, minor changes in oxygen requirement (below a level that was considered significant in the trial – see Respiratory Support in Supplementary Methods), and electrolyte imbalances on blood results. Foreseeable SAEs which could occur in our patient population were necrotising enterocolitis, intracranial abnormalities (haemorrhage, infarction or white matter damage), microbiologically confirmed or clinically suspected late-onset invasive infection, retinopathy of prematurity, patent ductus arteriosus, congenital abnormalities and death (if expected).

Statistical rationale and changes from the protocol

To account for multiple testing, we had intended to use Hochberg's procedure to control the family-wise error rate for the co-primary outcome measures, and to test secondary outcomes at a 1% significance level (see Protocol). In light of early trial cessation, this was revised to a 5% significance level for all outcomes (SAP, version 2.0). Also, as the sample size was smaller than anticipated due to early trial cessation, the statistical models were not adjusted for minimisation factors; the clinical stability analysis did not use poisson, logistic or linear regression models; and the safety analysis used risk differences instead of logistic regression. These changes are listed in the SAP version 2.0.

As this was an efficacy study the analysis was per protocol, as specified in the protocol. However, as all infants received the drug to which they had been randomised (except for one infant, withdrawn from the placebo arm prior to study), the per protocol analysis was equivalent to intention-to-treat analysis and the safety analysis was based upon the safety population, according to the treatment received.

Supplementary results

Stopping of the trial

As per protocol, the DMC convened for a review of the safety data after the recruitment of 25 infants. The stopping boundary was crossed as 3 of the 12 morphine-treated infants (25%) and none of the 13 placebo-treated infants (0%) had experienced apnoeas requiring resuscitation with NIPPV in the 24-hours after post-administration; a risk difference of 0.25, 80% CI 0.09 to 1.00, p=0.044. The DMC subsequently requested that the trial statisticians submit the data for all 31 infants that had been studied to date, to allow consideration of both the safety outcomes and the efficacy data for the co-primary outcomes. Analysis of the 31 infants randomised (only 30 studied as one was withdrawn) revealed that the stopping boundary had been crossed as 3 of the 15 morphine-treated infants (20%) had apnoeas requiring resuscitation with NIPPV in the 24-hours post-administration, compared with none of the 15 placebo-treated infants (0%); a risk difference of 0.20, 80% CI 0.05 to 1.00, p = 0.085. Following review of the clinical stability data and the co-primary outcomes, the DMC unanimously concluded that there was evidence of harm with no clear signals of benefit and recommended to the TSC that the trial should be suspended with immediate effect.

The independent members of the TSC concurred that the difference in safety event rates between the two groups crossed the predefined stopping boundary. They furthermore reviewed all the unblinded safety and efficacy data, and carefully considered the interpretation and wider implications of the results. The TSC was unable to recommend that the trial continue in its present form. They also unanimously concluded that they were unable to recommend trial cessation as to do so would mean the continued infliction of a necessary but painful and destabilising procedure upon preterm infants the world over without evidenced-based analgesia, and perpetuate a serious uncertainty in clinical practice. Instead the independent members of the TSC recommended that the trial should be paused while the investigators analysed the data collected in 31 infants, and considered possible revisions of the protocol, including a higher dose or dosage study with an expectation of adverse events and hence inclusion into the trial protocol of enhanced monitoring with provision of respiratory support should this be required. The Chair of the TSC communicated these recommendations to the funder (National Institute for Health Research - NIHR). NIHR were supportive of the TSC’s recommendations, but decided that the redesign of the trial would require a new funding application. NIHR withdrew funding but stated that they would welcome a new application for consideration given the importance of the clinical issue. The Central Monitoring Team stopped the Poppi trial on 15th March 2018, and the data from the 31 participants were analysed.

Supplementary Figure 1: Study timeline

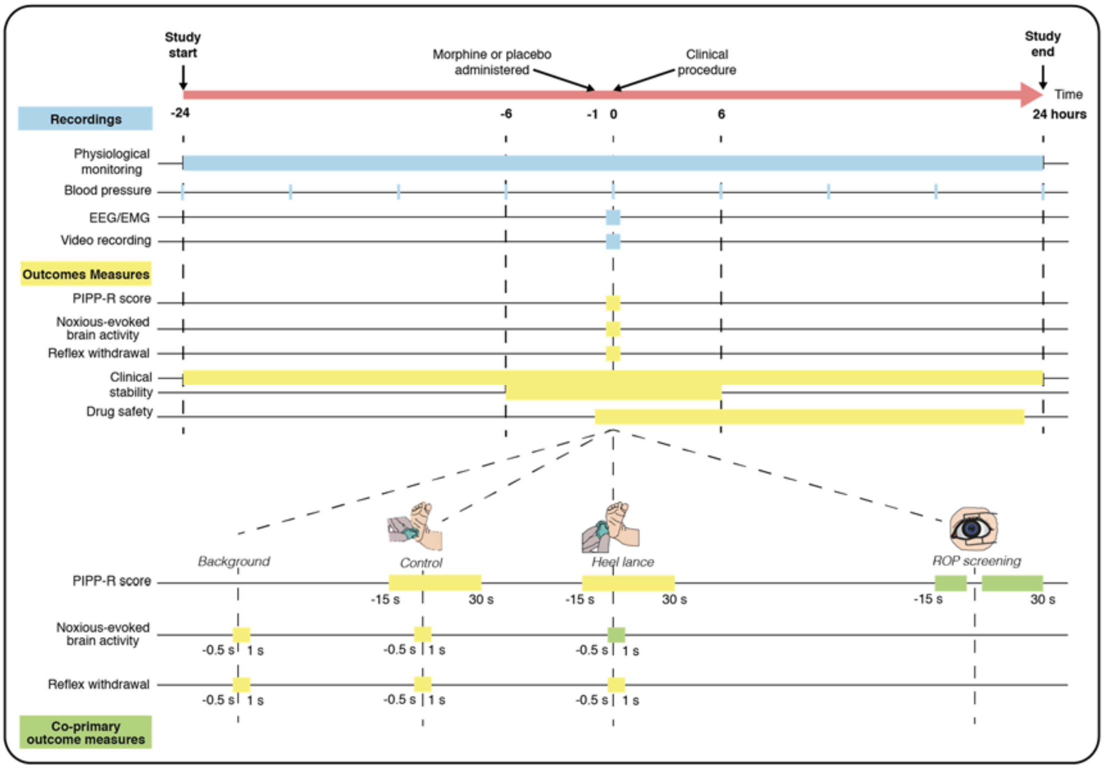

Supplementary Figure 2: Pain measures increase following the noxious clinical procedures

(A) Mean (SE) PIPP-R score in all infants following the control heel lance, heel lance, and the ROP screening. (B) Average EEG in background activity and following the control heel lance and heel lance in all infants. The (Woody-) filtered EEG is shown overlaid with the noxious-evoked brain activity template (in red) and the median (SE) magnitude of the noxious-evoked brain activity is shown in all 3 conditions. (C) Reflex withdrawal activity during a background period and following the control heel lance and heel lance in all infants. The root-mean-square (RMS) in 250ms windows is used to calculate the magnitude of the reflex withdrawal in all three conditions.

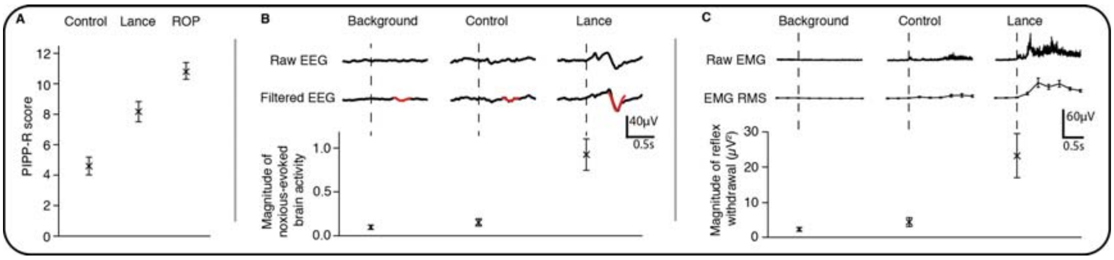

Supplementary Figure 3: Identification of episodes of bradycardia, oxygen desaturation and apnoea in infants

An example of an apnoeic event, with concurrent bradycardia and oxygen desaturation in an infant. Time 0 is the start of the bradycardia. (A) A bradycardia was defined as a period during which the heart rate dropped below 100 (grey dashed line) for at least 15 seconds. (B) A desaturation was defined as a period in which the oxygen saturation dropped below 80 (grey dashed line) for at least 10 seconds. (C) The ECG trace during this episode indicates a clear slowing of the heart rate. (D) Apnoeas were identified by reviewing the impedance pneumograph during an episode of bradycardia or from clinical notes. In this case, the apnoea was not recorded by the clinical team, but can clearly be observed from the impedance pneumograph as a pause in respiration lasting for at least 20 seconds. The apnoea starts approximately 18 seconds prior to the bradycardia. This apnoea is followed by a subsequent short pause in breathing between approximately 19 and 26 seconds after the bradycardia, which is not long enough to be defined as a subsequent apnoea.

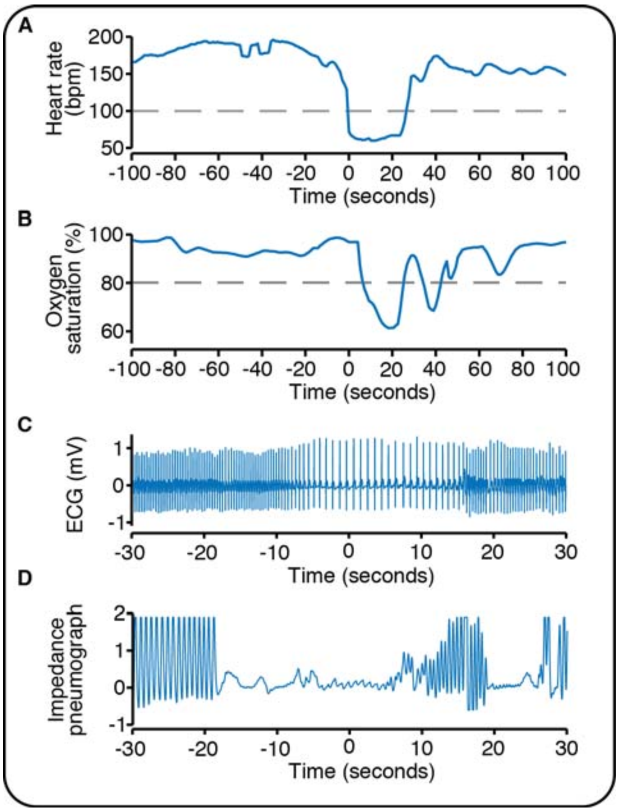

Supplementary Figure 4: Noxious-evoked brain activity in individual infants following clinical heel lance

Changes in brain activity at the Cz electrode following the heel lance are presented for each infant who received either morphine or placebo. The validated template of noxious-evoked brain activity (red) is shown overlaid on the EEG activity in the time window of interest (400–700 ms after the stimulus, grey shaded box). The green vertical lines indicate the point of stimulation.

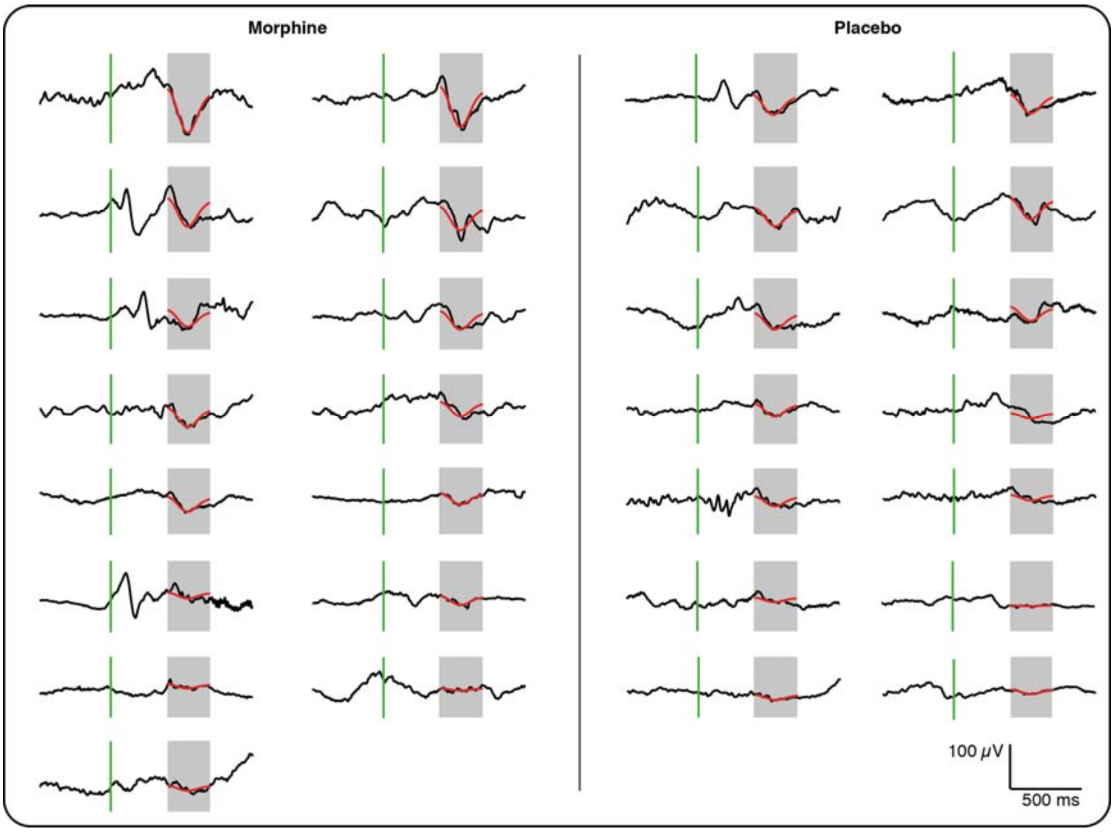

Supplementary Figure 5: Mean blood pressure

The average mean 6-hourly blood pressures are shown across the 48-hour monitoring period for infants who received placebo (blue) or morphine (green). Time 0 is the time of the clinical procedure. Error bars indicate mean ± standard error of the mean.

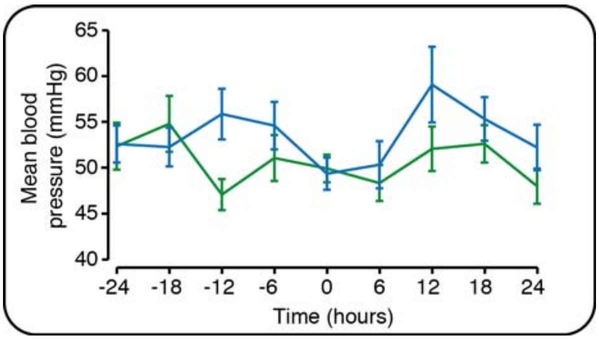

Supplementary Figure 6: Physiological data from each individual infant

48-hour traces of individual infant's heart rate and oxygen saturation are shown. Tachycardias, bradycardias, desaturation and apnoeas are indicated as red vertical lines, and the counts of these, split according to the 24 hours prior to the clinical procedure and 24 hours after the clinical procedure, are given on the left-hand side. Time 0 is the point of the clinical procedure.

Infants who received morphine

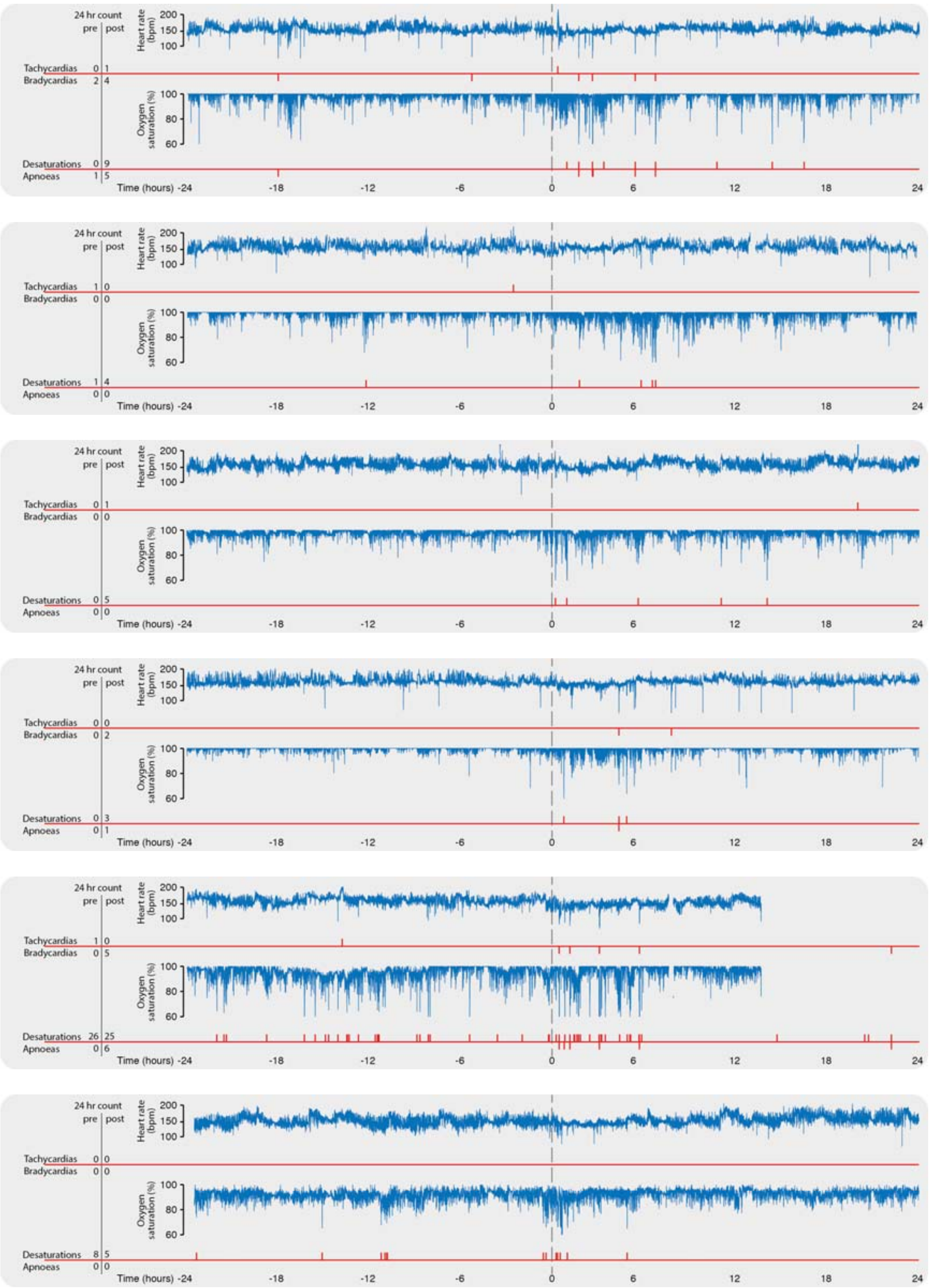

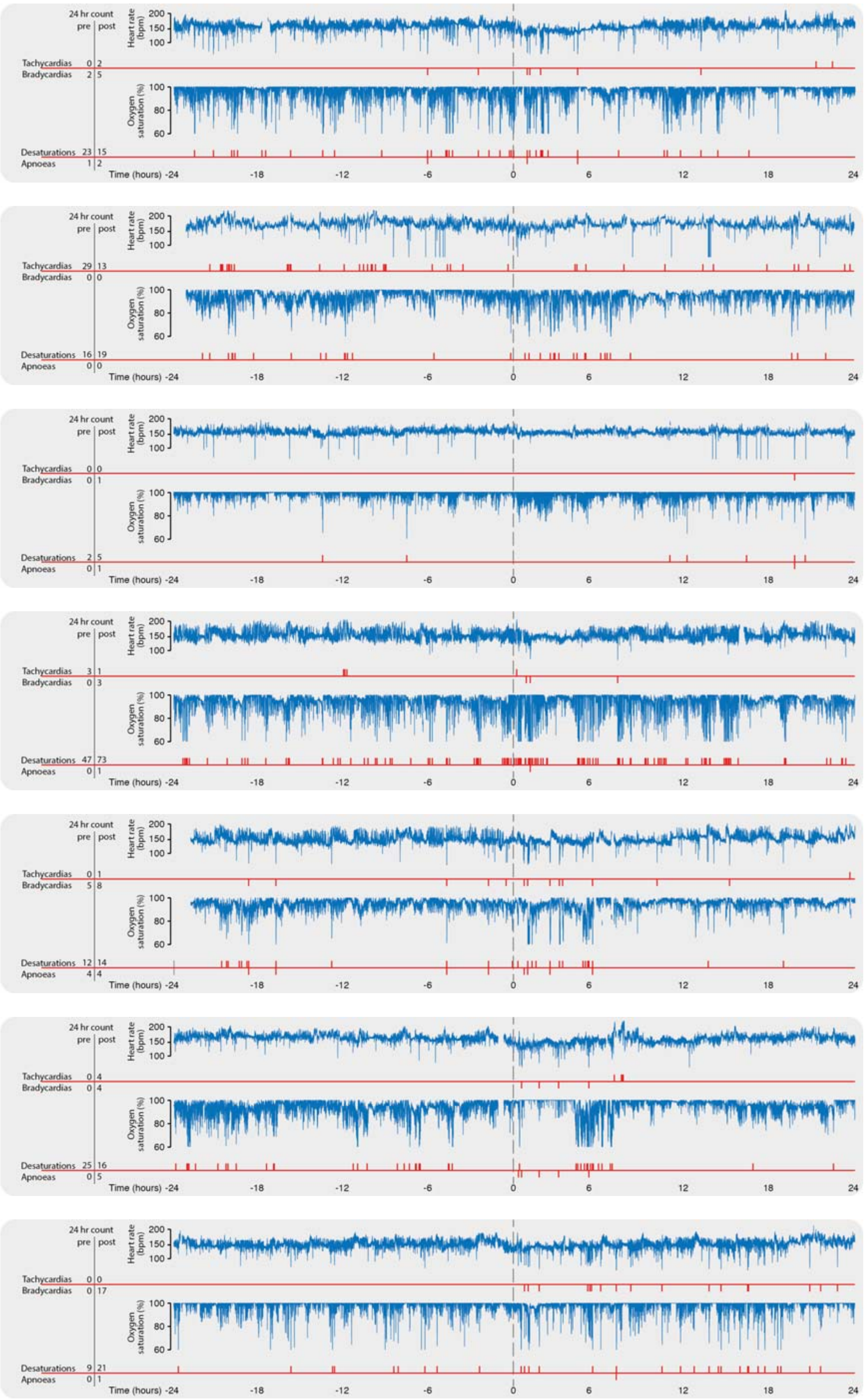
24 hr count
pre | post
Heart rate (bpm)
200
150
100
Tachycardias 0 | 2
Bradycardias 2 | 5
Oxygen saturation (%)
100
80
60
Desaturations 23 | 15
Apnoeas 1 | 2
Time (hours) -24 -18 -12 -6 0 6 12 18 24
24 hr count
pre | post
Heart rate (bpm)
200
150
100
Tachycardias 29 | 13
Bradycardias 0 | 0
Oxygen saturation (%)
100
80
60
Desaturations 16 | 19
Apnoeas 0 | 0
Time (hours) -24 -18 -12 -6 0 6 12 18 24
24 hr count
pre | post
Heart rate (bpm)
200
150
100
Tachycardias 0 | 0
Bradycardias 0 | 1
Oxygen saturation (%)
100
80
60
Desaturations 2 | 5
Apnoeas 0 | 1
Time (hours) -24 -18 -12 -6 0 6 12 18 24
24 hr count
pre | post
Heart rate (bpm)
200
150
100
Tachycardias 3 | 1
Bradycardias 0 | 3
Oxygen saturation (%)
100
80
60
Desaturations 47 | 73
Apnoeas 0 | 1
Time (hours) -24 -18 -12 -6 0 6 12 18 24
24 hr count
pre | post
Heart rate (bpm)
200
150
100
Tachycardias 0 | 1
Bradycardias 5 | 8
Oxygen saturation (%)
100
80
60
Desaturations 12 | 14
Apnoeas 4 | 4
Time (hours) -24 -18 -12 -6 0 6 12 18 24
24 hr count
pre | post
Heart rate (bpm)
200
150
100
Tachycardias 0 | 4
Bradycardias 0 | 4
Oxygen saturation (%)
100
80
60
Desaturations 25 | 16
Apnoeas 0 | 5
Time (hours) -24 -18 -12 -6 0 6 12 18 24
24 hr count
pre | post
Heart rate (bpm)
200
150
100
Tachycardias 0 | 0
Bradycardias 0 | 17
Oxygen saturation (%)
100
80
60
Desaturations 9 | 21
Apnoeas 0 | 1
Time (hours) -24 -18 -12 -6 0 6 12 18 24

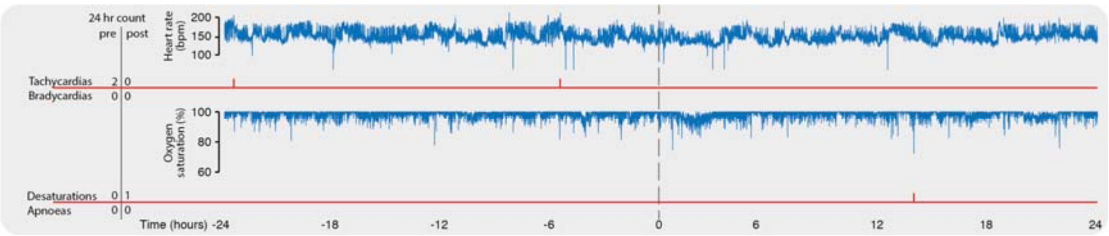

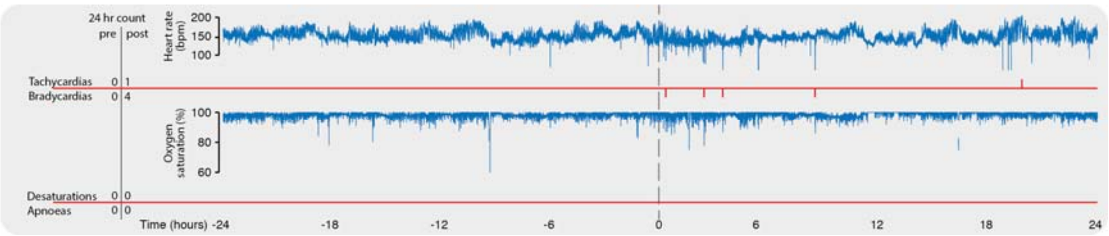

Infants who received placebo:

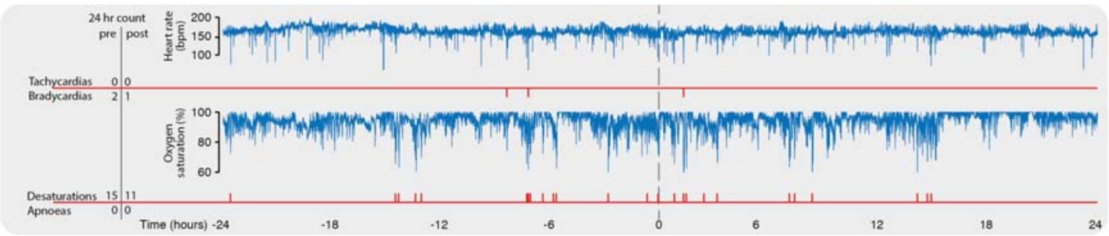

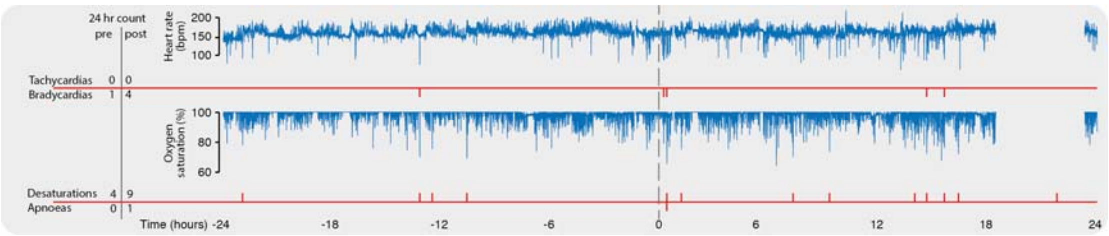

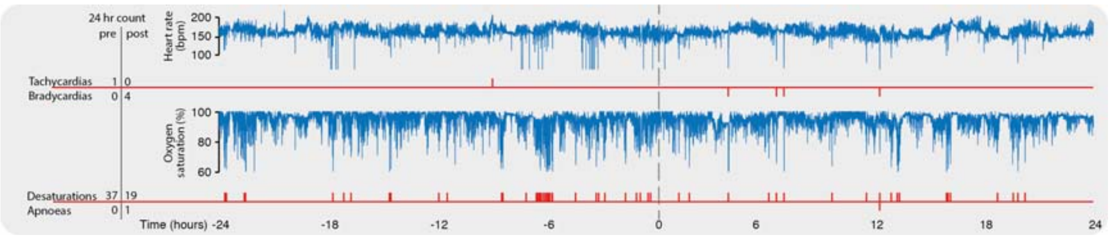

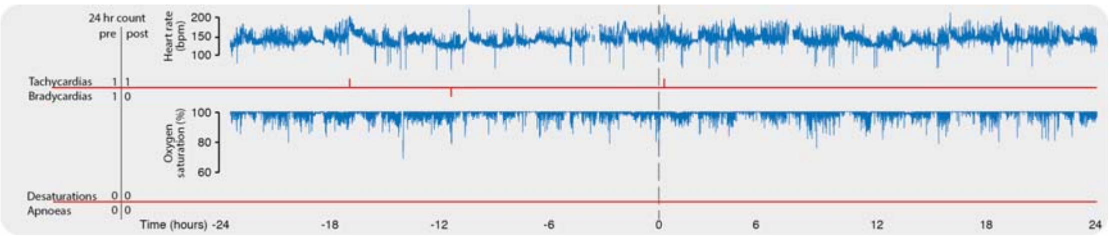

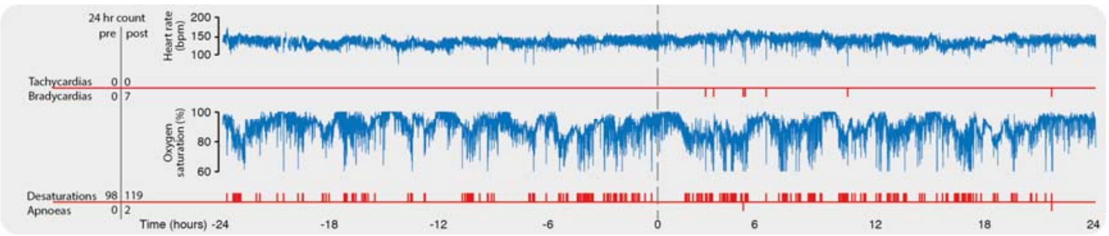

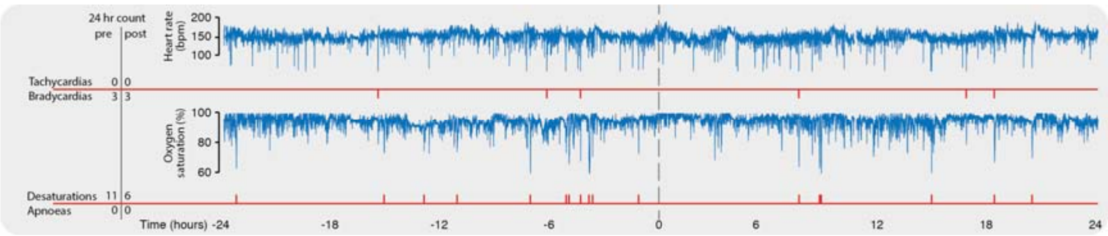
24 hr count
pre | post
Heart rate (bpm)
200
150
100
Tachycardias 0 0
Bradycardias 3 3
Oxygen saturation (%)
100
80
60
Desaturations 11 6
Apnoeas 0 0
Time (hours) -24 -18 -12 -6 0 6 12 18 24

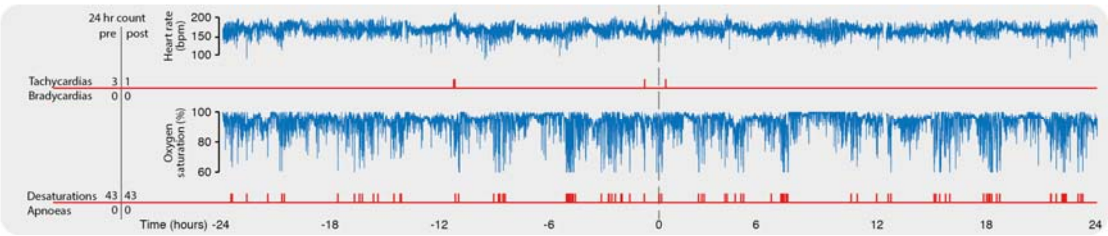
24 hr count
pre | post
Heart rate (bpm)
200
150
100
Tachycardias 3 1
Bradycardias 0 0
Oxygen saturation (%)
100
80
60
Desaturations 43 43
Apnoeas 0 0
Time (hours) -24 -18 -12 -6 0 6 12 18 24

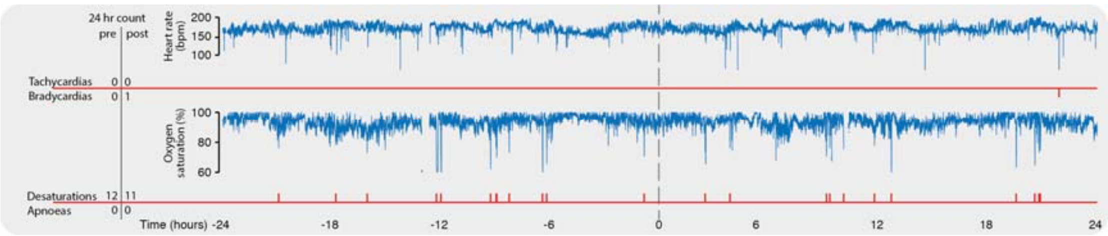
24 hr count
pre | post
Heart rate (bpm)
200
150
100
Tachycardias 0 0
Bradycardias 0 1
Oxygen saturation (%)
100
80
60
Desaturations 12 11
Apnoeas 0 0
Time (hours) -24 -18 -12 -6 0 6 12 18 24

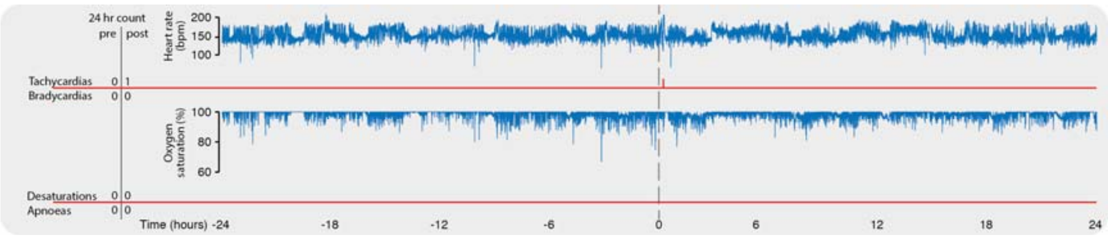
24 hr count
pre | post
Heart rate (bpm)
200
150
100
Tachycardias 0 1
Bradycardias 0 0
Oxygen saturation (%)
100
80
60
Desaturations 0 0
Apnoeas 0 0
Time (hours) -24 -18 -12 -6 0 6 12 18 24

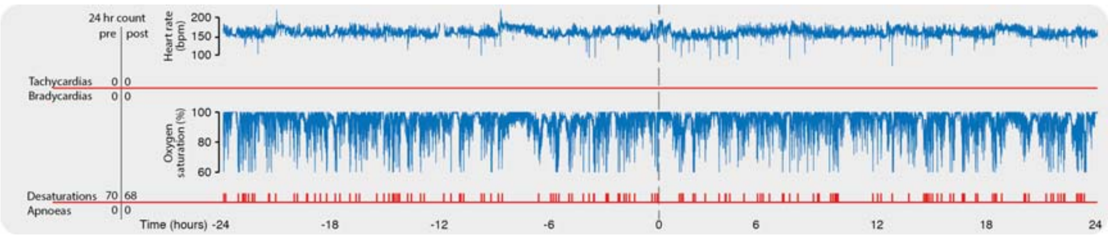
24 hr count
pre | post
Heart rate (bpm)
200
150
100
Tachycardias 0 0
Bradycardias 0 0
Oxygen saturation (%)
100
80
60
Desaturations 70 68
Apnoeas 0 0
Time (hours) -24 -18 -12 -6 0 6 12 18 24

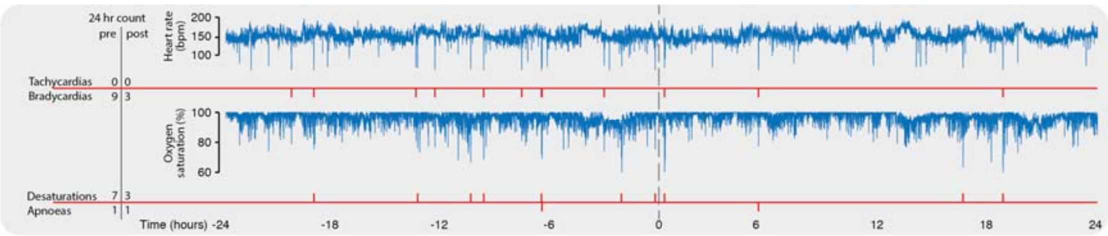
24 hr count
pre | post
Heart rate (bpm)
200
150
100
Tachycardias 0 0
Bradycardias 9 3
Oxygen saturation (%)
100
80
60
Desaturations 7 3
Apnoeas 1 1
Time (hours) -24 -18 -12 -6 0 6 12 18 24

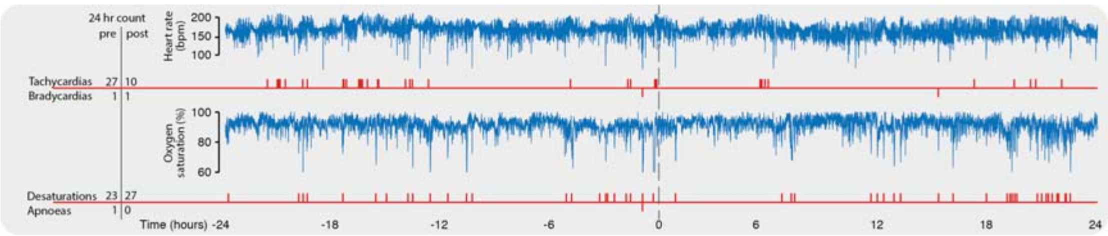
24 hr count
pre | post
Heart rate (bpm)
200
150
100
Tachycardias 27 10
Bradycardias 1 1
Oxygen saturation (%)
100
80
60
Desaturations 23 27
Apnoeas 1 0
Time (hours) -24 -18 -12 -6 0 6 12 18 24

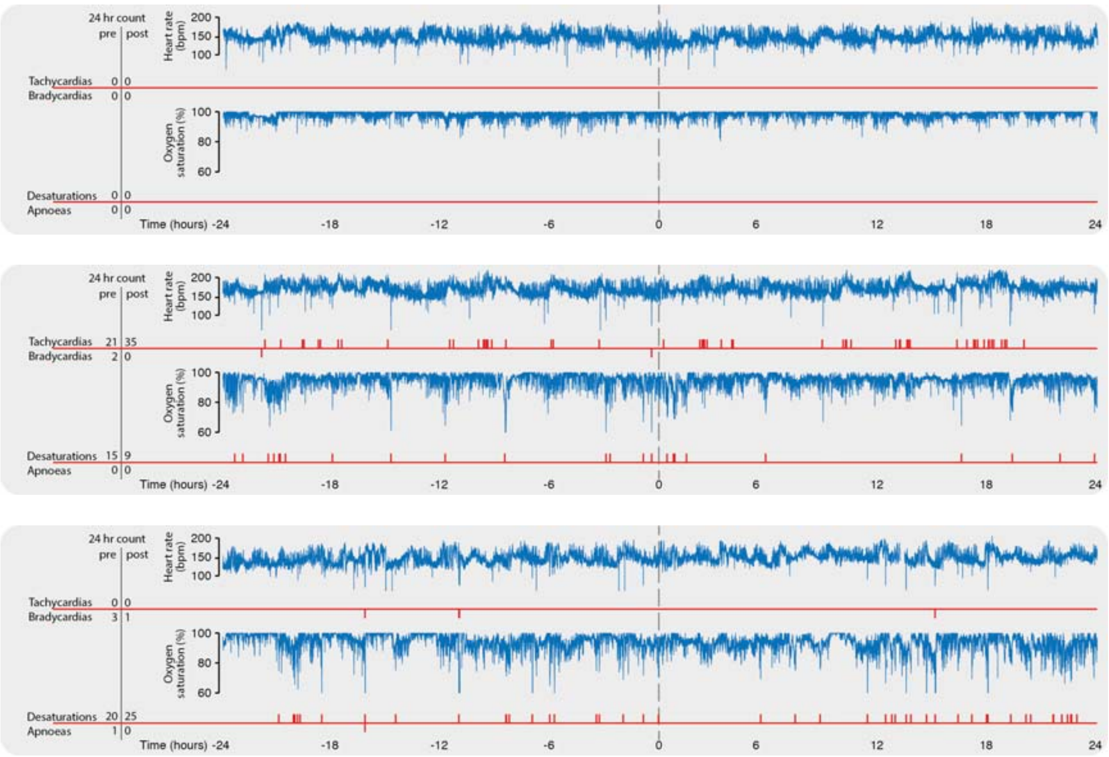
24 hr count
pre | post
Heart rate (bpm)
200
150
100
Tachycardias 0 0
Bradycardias 0 0
Oxygen saturation (%)
100
80
60
Desaturations 0 0
Apnoeas 0 0
Time (hours) -24 -18 -12 -6 0 6 12 18 24
24 hr count
pre | post
Heart rate (bpm)
200
150
100
Tachycardias 21 35
Bradycardias 2 0
Oxygen saturation (%)
100
80
60
Desaturations 15 9
Apnoeas 0 0
Time (hours) -24 -18 -12 -6 0 6 12 18 24
24 hr count
pre | post
Heart rate (bpm)
200
150
100
Tachycardias 0 0
Bradycardias 3 1
Oxygen saturation (%)
100
80
60
Desaturations 20 25
Apnoeas 1 0
Time (hours) -24 -18 -12 -6 0 6 12 18 24

Supplementary References

1. Worley A, Fabrizi L, Boyd S, Slater R. Multi-modal pain measurements in infants. J Neurosci Methods,. 2012;205(2):252-7.
2. Stevens BJ, Gibbins S, Yamada J, Dionne K, Lee G, Johnston C, et al. The premature infant pain profile-revised (PIPP-R): initial validation and feasibility. Clin J Pain,. 2014;30(3):238-43.
3. Hartley C, Duff EP, Green G, Mellado GS, Worley A, Rogers R, et al. Nociceptive brain activity as a measure of analgesic efficacy in infants. Sci Transl Med. 2017;9(388).
4. Hartley C, Goksan S, Poorun R, Brotherhood K, Mellado GS, Moultrie F, et al. The relationship between nociceptive brain activity, spinal reflex withdrawal and behaviour in newborn infants. Sci Rep,. 2015;5.
5. Maris E, Oostenveld R. Nonparametric statistical testing of EEG- and MEG-data. J Neurosci Methods,. 2007;164(1):177-90.
